# Physiological–Biochemical Characteristics and a Transcriptomic Profiling Analysis Reveal the Postharvest Wound Healing Mechanisms of Sweet Potatoes under Ascorbic Acid Treatment

**DOI:** 10.3390/foods13162569

**Published:** 2024-08-17

**Authors:** Hongxia Xuan, Jiyu Cheng, Linjiang Pang, Liqing Yin, Yuge Guan, Junfeng Cheng, Xinghua Lu, Guoquan Lu

**Affiliations:** 1 College of Food and Health, Zhejiang A&F University, Hangzhou 311300, China; xuan9947@163.com (H.X.); ljpang@zafu.edu.cn (L.P.); ylqing@zafu.edu.cn (L.Y.); gyg@zafu.edu.cn (Y.G.); 20220071@zafu.edu.cn (J.C.); xhlu@zafu.edu.cn (X.L.); 2 Institute of Root & Tuber Crops, Zhejiang A&F University, Hangzhou 311300, China; lugq10@zju.edu.cn

**Keywords:** sweet potato, ascorbic acid, wound healing, lignin content, disease resistance, genes

## Abstract

Sweet potatoes are extremely vulnerable to mechanical wounds during harvesting and postharvest handling. It is highly necessary to take measures to accelerate wound healing. The effect of 20 g L^−1^ of ascorbic acid (AA) treatment on the wound healing of sweet potatoes and its mechanisms were studied. The results validated that AA treatment significantly reduced the weight loss rate and disease index. AA treatment effectively enhanced the formation speed of lignin and SPP at the wound sites, decreased the MDA content, and maintained the cell membrane integrity. AA enhanced the activities of PAL, C4H, 4CL, CAD, and POD and increased the contents of chlorogenic acid, caffeic acid, sinapic acid, ferulic acid, cinnamic acid, *p*-coumaryl alcohol, sinapyl alcohol, coniferyl alcohol, and lignin. Based on a transcriptomic analysis, a total of 1200 genes were differentially expressed at the sweet potato wound sites by the AA treatment, among which 700 genes were upregulated and 500 genes were downregulated. The KEGG pathway analysis showed that the differentially expressed genes were mainly involved in phenylalanine, tyrosine, and tryptophan biosynthesis; phenylpropanoid biosynthesis; and other wound healing-related pathways. As verified by a qRT-PCR, the AA treatment significantly upregulated the gene expression levels of *IbSKDH*, *IbADT/PDT*, *IbPAL*, and *Ib4CL* at the wound sties.

## 1. Introduction

Sweet potato (*Ipomoea batatas* (L.) Lam) is an important food crop that ensures food safety and is a competitive energy crop. It has high yield, high adaptability, and a wide application. According to the statistics of the FAO, in 2022, the planting area of sweet potatoes in China was about 2.2 million hectares, with an annual production of about 47 million tons, accounting for approximately 29.8% and 54.2% of the global total, respectively. Sweet potato is rich in starch, dietary fiber, carotene, minerals, and other nutrients. It also contains phenolic acids, flavonoids, anthocyanins, and other bioactive ingredients with antioxidant, hypoglycemic, and antiaging effects. In addition to being used as fresh food, sweet potato is often processed into various foods as raw and auxiliary material [1]. Because of the thin skin and tender flesh, sweet potato is inevitably prone to wounding stress during harvesting and transportation. Various wounds not only accelerate water loss but also provide the channel for invasion by pathogens such as *Ceratocystis fimbriata* and *Rhizopus stolonifera*. During storage, the wounds can lead to diseases such as black rot and soft rot sweet potato [2], ultimately resulting in severe losses [3]. About 15% of sweet potatoes in China are infected by pathogens due to improper storage every year, and the decay rate caused by pathogen infection due to mechanical damage accounts for more than 50% of the total loss [4].

Wound healing treatment can help maintain the quality of various fruits and vegetables after harvest and help them resist pathogen infection. As is well known, under the appropriate environmental conditions, sweet potato wounds can initiate self-healing after harvest, naturally form healing tissue, and enhance disease resistance [5]. However, the natural healing of sweet potatoes usually takes approximately 2–3 weeks. During the long-term healing process, the quality of sweet potatoes can easily deteriorate due to external factors [6]. Therefore, it is necessary to take additional measures to shorten the healing time. At present, healing can be accelerated through heat treatment or chemical treatment. Heat treatment, such as through hot air, hot water, or microwave treatment, is conducive to wound healing in sweet potatoes [7], but given the need for the corresponding heating equipment during processing, there are several problems in practical application, such as inconvenient operation, high energy consumption, and non-uniform heating. Previous studies have shown that suitable concentrations of various substances, such as ethylene [8], and benzothiazole (BTH) [5], can also promote healing. Chemical methods are easier to carry out and require relatively less equipment, making them easier to apply. It is still unclear whether chemical agents will cause residue and environmental pollution issues. However, it is necessary to further explore greener, safer, and more efficient wound healing methods.

Ascorbic acid (AA) is a natural and strong antioxidant [9]. It is usually used for maintaining freshness and color, as well as reducing weight loss and the disease index of postharvest fruits and vegetables [10,11,12]. AA degrades H_2_O_2_ through the ASA-GSH cycle in cooperation with glutathione (GSH) and antioxidant enzymes, thus reducing cell damage [13]. It has been reported that AA can participate in the regulation of lignin biosynthesis and the regulation of xylem metabolism. AA is involved in controlling the polymerization of phenolic compounds, which is highly important for the regulation of lignin biosynthesis [14]. AA catalyzes the production of the hydroxyproline-rich glycoproteins [15] necessary for cell expansion and division in the endoplasmic reticulum and participates in phenoxy radical-mediated cross-linking of cell wall components, resulting in cell wall solidification [16]. Coban et al. found that exogenous AA treatment could increase the xylem vessel diameter and induce lignification in cucumber seedlings, indicating that AA treatment promoted the formation of xylem and the lignification of cortical cells [17].

In previous studies, AA has been widely used for the preservation of fruits and vegetables, but the effect of AA treatment on the postharvest wound healing of sweet potatoes and its related mechanisms have not been reported. Therefore, we studied the possible physiological and molecular mechanisms by which AA promotes sweet potato wound healing by mainly focusing on (1) observing the deposition of lignin and suberin in sweet potato healing tissues, (2) determining the effects of AA treatment on the phenylpropane metabolite content and key enzyme activities in the healing tissue, and (3) screening the differentially expressed genes through transcriptomic sequencing and exploring the key genes related to wound healing.

## 2. Materials and Methods

### 2.1. Sweet Potato Roots

The sweet potato cultivar used was ‘Xinxiang’, which was harvested at the Institute of Potato Crops of Zhejiang A & F University in October 2023. The selected sweet potatoes had a uniform shape and size without mechanical damage and disease.

Based on the results of the previous screening (Figure S1), 20 g L^−1^ of AA (Yuanye, Shanghai, China) was prepared for use.

*Ceratocystis fimbriata* Ellis & Halsted (*C. fimbriata*) was obtained from the Xuzhou Institute of Agricultural Sciences, Xuhuai region, Jiangsu Province.

### 2.2. Artificial Wounding

The artificial wounding was performed as described by Xue et al. [18]. The sweet potatoes were washed, disinfected with 1% NaClO solution for 3 min, rinsed with distilled water, and dried. A stainless steel scraper disinfected with 75% ethanol was used to evenly cut three wounds (20 × 10 × 2 mm, length × width × depth) on the equatorial part of the sweet potato surface.

### 2.3. AA Treatment and Wound Healing

The artificially wounded sweet potatoes were randomly divided into two groups. One group was soaked in 20 g L^−1^ of AA for 10 min, and the other group was soaked in distilled water for 10 min as a control group (the CK group). After natural drying, all the sweet potatoes were allowed to heal in the dark for 7 d at room temperature (25 ± 1 °C, RH85-90%). Both treatments had three replicates, and each replicate comprised 100 roots.

### 2.4. Sampling

The sampling was performed as described by Wang et al. [5] with minor modifications. At 0, 1, 3, 5, and 7 d of healing, a sterile scalpel was used to collect a 2 mm thick healing tissue sample, which was rapidly frozen in liquid nitrogen, powdered using a grinder (IKA A11 basic, IKA, Staufen, Germany), and stored at −80 °C for the subsequent analysis.

### 2.5. Determination of Weight Loss Rate

A gravimetric method was used to determine the weight loss rate of the healing roots after 0, 1, 3, 5, and 7 d.

### 2.6. Determination of Disease Index

The disease index was measured as described by Zhu et al. [19] with minor modifications. *C. fimbriata* spore suspensions at a concentration of 1 × 10^6^ spores mL^−1^ were prepared according to the procedure described by Wu [20]. At 0, 1, 3, 5, and 7 d of healing, 20 µL of spore suspension was evenly applied to the surface of each sweet potato wound and allowed to dry. Then, the samples were stored in the dark at room temperature for the disease index analysis. The calculation formula was as follows:

Disease index = (∑(number of wounds × grade of disease))/(highest grade disease × total wounds) × 100

The grade of disease was defined as 0 (no incidence), 1 (one-quarter wound area incidence), 2 (one-half wound area incidence), 3 (three-quarter wound area incidence), or 4 (full wound area incidence).

### 2.7. Determination of Firmness

The firmness was determined as described by Soteriou et al. [21] with slight modifications. At 0, 1, 3, 5, and 7 d of healing, the tissue (10 × 10 × 5 mm, length × width × depth) was cut perpendicularly to the wound on the sweet potato with a scalpel. The firmness was measured by a texture analyzer (TMS-PRO, FTC, Washington, DC, USA) with a 5 mm diameter probe. These results for firmness are represented as N. The measurement parameters were as follows: the pretest speed was 30 mm min^−1^, the test speed was 60 mm min^−1^, the posttest speed was 90 mm min^−1^, the compression strain was 60%, the pause time was 2 s, and the trigger force was 0.2 N.

### 2.8. Staining and Microscopy

The lignin deposition was observed according to the description reported by He et al. [22]. Slices that were 0.2–0.3 mm thick were cut from the wound surfaces, and then washed with distilled water 3 times for observation. The slices were stained with 1% (*w*/*v*) of phloroglucinol–hydrochloric acid solution [6], and then the lignin accumulation was observed and imaged using an optical microscope (SW380T, Swift, New York, NY, USA) at a magnification of 10 × 10.

The suberin polyphenol (SPP) deposition was examined as described by Fugate et al. [23]. Slices that were 0.2–0.3 mm thick and approximately 1 cm in length and width were cut from the wound surface. Based on autofluorescence, the SPP accumulation deposition was observed and photographed using a fluorescence microscope (Leica DM4B, Leica, Wetzlar, Germany).

### 2.9. Determination of Total Phenol, Total Flavonoid, and Lignin Contents

A 1.0 g sample was accurately weighed out, 6 mL of 80% methanol was added, and it was then extracted for 10 min by an ultrasonic instrument (at an ultrasonic frequency of 40 KHz), followed by centrifugation at 12,000× *g* for 10 min. The total phenol and total flavonoid content were determined as described by Torres et al. [24] and Xie et al. [25], respectively.

The lignin content was determined by a commercial kit (Beijing Boxbio Science & Technology Co., Ltd., Beijing, China). A 2.0 g sample was weighed out, dried to a constant weight at 80 °C, ground through a 40-mesh sieve, and then quantified according to the instructions. At the end of the reaction, the absorbance was measured at 280 nm. The lignin content was presented as g kg^−1^ of dry weight (DW).

### 2.10. Determination of Individual Phenolic Acid and Lignin Monomer Contents

The separation and quantification of the individual phenolic acid and lignin monomer contents in the healing tissues of the sweet potatoes were performed using high-performance liquid chromatography (HPLC) (LC-20A, Shimadzu Corporation, Kyoto, Japan), according to the procedure described by Ayaz et al. [26] with minor modifications. A 2.0 g sample powder was homogenized with 4 mL of 80% methanol and extracted for 10 min by an ultrasonic instrument (at an ultrasonic frequency of 40 KHz), centrifuged at 12,000× *g* for 20 min, then the supernatant was filtered through a 0.22 μm membrane prior to analysis by the HPLC. The chromatographic column used was a C18 reversed-phase chromatographic column (250 × 4.6 mm, 5 μm). The mobile phase consisted of 80% methanol (A) and a 0.5% acetic acid solution (B). The elution gradient was as follows: 0–3 min, 5% A; 3–15 min, 8% A; 15–25 min, 25% A; 25–35 min, 28% A; 35–42 min, 40% A; 42–45 min, 5% A; 45–50 min, 100% A; and 50–60 min, 5% A. The column temperature, flow rate, and injection volume were set at 40 °C, 1 mL min^−1^, and 10 μL, respectively. Chlorogenic acid was detected at 326 nm, caffeic acid and sinapic acid at 325 nm, *p*-coumaryl acid at 310 nm, ferulic acid and sinapyl alcohol at 322 nm, coniferyl alcohol at 263 nm, *p*-coumaryl alcohol at 273 nm, and cinnamic acid at 276 nm. The standard curve equations for each phenolic acid were as follows: chlorogenic acid, y = 31,944x − 12,284, R^2^ = 0.9982; caffeic acid, y = 60,669x − 14,720, R^2^ = 0.9992; sinapic acid, y = 58,370x − 7553, R^2^ = 0.9993; *p*-coumaryl acid, y = 100,804x − 15,740, R^2^ = 0.9994; ferulic acid, y = 67,336x − 20,029, R^2^ = 0.9991; sinapyl alcohol, y = 13,732x − 2383.7, R^2^ = 0.9995; coniferyl alcohol, y = 46,488x − 4521.7, R^2^ = 0.9997; *p*-coumaryl alcohol, y = 51,329x − 8579.3, R^2^ = 0.9995; and cinnamic acid, y = 121,150x + 67,822, R^2^ = 0.9993. According to the standard curves, the contents were calculated and expressed as g kg^−1^. The standard chart of the phenolic acids is shown in Figure S2.

### 2.11. Determination of the Malondialdehyde (MDA) Content

The measurement process followed the method of Jiang et al. [27]. A 1.0 g sample was accurately weighed out and mixed evenly with 8.0 mL of precooled 10% trichloroacetic acid (TCA) solution, followed by extraction for 30 min in an ice bath and centrifugation for 20 min at 4 °C and 10,000× *g*. Then, 2.0 mL of 0.67% 2-thiobarbituric acid (TBA) solution was added to 2.0 mL of the supernatant, and the reaction was performed for 20 min in a boiling water bath. After cooling the reaction solution, the absorbances at 600 nm, 450 nm, and 532 nm were simultaneously measured.

### 2.12. Determination of the Permeability of Cell Membranes

The measurement process followed the method of Jiang et al. [27]. Five circular pieces of uniformly sized healing tissue were taken with a punch, rinsed three times with deionized water, and incubated in 30 mL of deionized water for 1 h at 35 °C, before the initial conductivity E_0_ and the final conductivity E_1_ were recorded. Then, the samples were incubated in a boiling water bath for 0.5 h, and the conductivity was determined as E_2_ after cooling. The calculation formula was as follows:

Permeability of cell membranes = (E_1_ − E_0_)/E_2_ × 100%

### 2.13. Determination of PAL, C4H, 4CL, Cinnamyl Alcohol Dehydrogenase (CAD), and POD Activities

The PAL activity was determined as described by Qu et al. [28]. The C4H and 4CL activities were analyzed according to the method of Wang et al. [29]. The CAD activity was performed as described by Li et al. [30]. The POD activity was detected as described by Li et al. [31]. The activities of PAL, C4H, 4CL, CAD, and POD were expressed as U kg^−1^, where U = 0.01 OD290 h^−1^, U = 0.01 OD340 min^−1^, U = 0.01 OD320 min^−1^, U = 0.01 OD340 min^−1^, and U = 0.01 OD470 min^−1^, respectively.

### 2.14. RNA Sequencing

The total RNA was extracted from the healing tissues of the sweet potato on day 3 using a Plant RNA Purification Reagent (Invitrogen, Carlsbad, CA, USA) kit. There were 3 biological replicates in each treatment. The concentration and integrity of the RNA were determined by a Nanodrop 2000 spectrophotometer (Thermo Fisher Scientific, Waltham, MA, USA) and agarose gel electrophoresis (DYY-6C, Beijing Liuyi Biotechnology CO.,Ltd, China). After the RNA samples passed the quality tests, the library construction and RNA sequencing were completed by Shanghai Meiji Biological Co., Ltd. (Shanghai, China). The clean data of each sample were compared with the reference genome of wild sweet potato (*Ipomoea trifida*) (http://sweetpotato.plantbiology.msu.edu/index.shtml, accessed on 9 February 2024). The alignment of the transcriptome sequencing data with the reference genome is shown in Table S1. The differentially expressed genes (DEGs) between the two treatment groups were analyzed by the DESeq2 software (version 1.20.0). The screening criteria for identifying the DEGs were a *p* value < 0.05 and an FC ≥ 1.5. The DEGs were analyzed by GO function analysis and KEGG pathway enrichment analysis.

### 2.15. Quantitative Real-Time PCR (qRT-PCR)

The RNA-seq results were verified by a qRT-PCR. The cDNA was synthesized with a HiScript Q RT SuperMix for qRT-PCR (+gDNA wiper) kit (Vazyme, Nanjing, China) according to the manufacturer’s instructions. The housekeeping gene Ibtubulin was used as the reference gene, and ChamQ SYBR Color qPCR Master Mix (2×) reagent (Vazyme, Nanjing, China) and ABI7300 fluorescence quantitative PCR (Applied Biosystems, Waltham, MA, USA) were used for the analysis. The reaction mixture contained the following components: 10 µL of 2× ChamQ SYBR Color qPCR Master Mix, 0.8 μL each of the forward primer and reverse primer (5 µM), 0.4 µL of 50× ROX Reference Dye I, 2 µL of cDNA, and 6 µL of ddH_2_O. The qRT-PCR conditions were as follows: 95 °C for 5 min, followed by 40 cycles of 95 °C for 5 s, 55 °C for 30 s, and 72 °C for 40 s. *IbSKDH* (itf13g19040.t1), *IbADT/PDT* (itf04g31570.t1), *IbPAL* (itf06g07070.t1), and Ib4CL (itf03g10110.t1) were selected for the qRT-PCR verification. The relative expression levels of the genes were calculated by the comparative CT (2^−∆∆CT^) method. The primers used are listed in Table S2.

### 2.16. Statistical Analysis

Both treatment experiments were repeated three times. All the measurement results at each time point were expressed as the mean ± standard deviation (SD) using Microsoft Excel 2010. The significance of the differences between the samples was analyzed by a one-way analysis of variance (ANOVA), followed by Duncan’s multiple range tests at the 5% level (*p* < 0.05) and the 1% level (*p* < 0.01) using IBM SPSS Statistics 26.0. The correlation analysis was carried out using the correlation heatmap tool in HiPlot (https://hiplot.com.cn, accessed on on 9 February 2024).

## 3. Results

### 3.1. Weight Loss Rate, Disease Index, Firmness, and Appearance Changes

**Figure 1:**
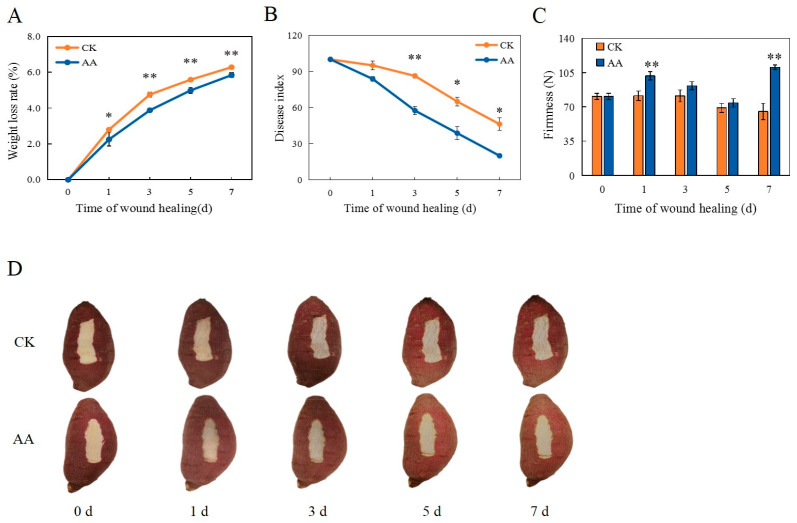
Weight loss rate (**A**), Disease index (**B**), Firmness (**C**), and Appearance changes (**D**) at the sweet potato wound sites during healing. The bars indicate the standard deviation (±SD). The asterisks indicate significant differences between the different treatments at the same time (* *p* < 0.05, ** *p* < 0.01).

During the healing period, the weight loss continued to increase gradually in the CK group and the AA-treated group, but the weight loss of the AA-treated sweet potatoes was significantly slowed down (Figure 1A). On days 3, 5, and 7, the weight loss rates of the AA-treated sweet potatoes were 4.8%, 5.6%, and 6.2%, respectively, which were significantly lower than those of the CK group.

AA treatment can clearly decrease the disease index (Figure 1B). After 3 d of healing, the disease index of the AA-treated sweet potatoes was extremely significantly lower than that of the CK group (by 33.3%). After 5 and 7 d of healing, the disease indices of the sweet potatoes treated with AA were significantly reduced, being 40.4% and 56.8% lower than the CK group, respectively.

During healing, the overall firmness of the sweet potatoes treated with AA was higher than that of the CK group. On days 1 and 7, the firmness of the AA-treated group was extremely significantly higher than that of the CK group (by 20.0% and 40.9%, respectively) (Figure 1C). It is indicated that AA treatment can enhance firmness, with high fruit firmness being beneficial for resisting pathogen infection [32].

The appearance changes of the sweet potatoes during the healing period were shown in Figure 1D. As a result of the AA treatment, the healing tissues formed at the wound site surface appeared smoother, whiter, and more complete during the healing period. However, browning and some obvious cracks were observed at the wound sites in the CK group. Meanwhile, the skin of the sweet potatoes with the AA treatment showed a brighter color than that of the control. The results showed that AA can help to form healing tissue and maintain a better color of the wound of the sweet potatoes during healing.

### 3.2. The Accumulation of Lignin and SPP

**Figure 2:**
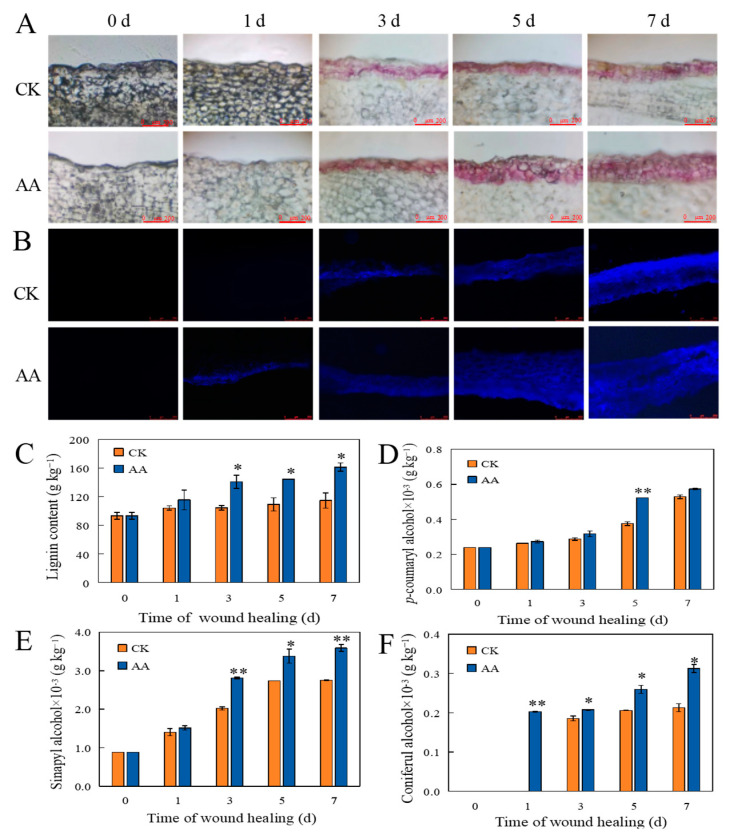
The accumulation of lignin (**A**) and SPP (**B**), Lignin content (**C**), *p*-coumaryl alcohol content (**D**), Sinapyl alcohol content (**E**), and Coniferyl alcohol content (**F**) at the sweet potato wound sites during healing. The bars indicate the standard deviation (±SD). The asterisks indicate significant differences between the different treatments at the same time (* *p* < 0.05, ** *p* < 0.01).

During the healing period, the red lignin in the sweet potato wounds increased continuously (Figure 2A). Red lignin deposition could be observed in the AA treatment group on the first day of healing. Compared with the CK group, the AA treatment significantly increased the thickness of the lignified cell layer in the sweet potato wounds during the same healing period. Meanwhile, during the healing period, the blue autofluorescence in the sweet potato wounds increased gradually. The deposition level of SPP was clearly observed in the wound site of the sweet potatoes treated with AA on the first day of healing. The SPP accumulation level after the AA treatment was significantly higher than that of the CK group with the increase in healing time (Figure 2B).

### 3.3. Total Lignin, Phenol, and Flavonoid Contents

**Figure 3:**
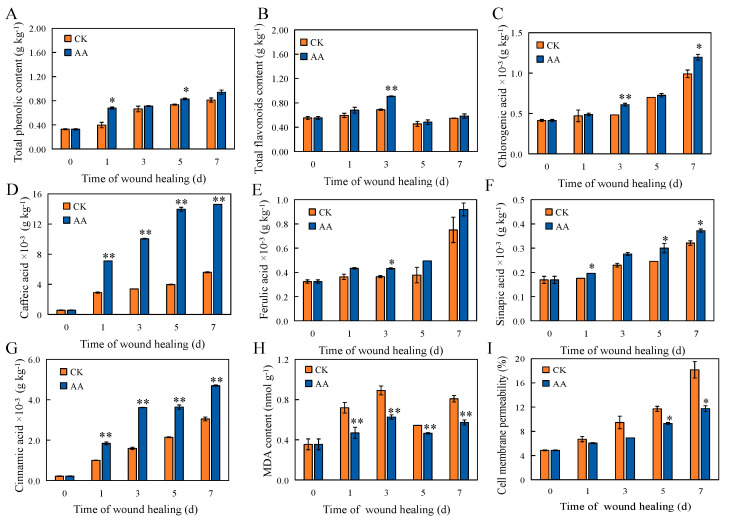
Total phenol content (**A**), Total flavonoid content (**B**), Chlorogenic acid content (**C**), Caffeic acid content (**D**), Ferulic acid content (**E**), Sinapic acid content (**F**), Cinnamic acid content (**G**), MDA content (**H**), and Cell membrane permeability (**I**) at the sweet potato wound sites during healing. The bars indicate the standard deviation (±SD). The asterisks indicate significant differences between the different treatments at the same time (* *p* < 0.05, ** *p* < 0.01).

The lignin content was significantly increased by the AA treatment at the sweet potato wound sites throughout the whole healing period (Figure 2C). On days 3, 5, and 7, the lignin content of the AA-treated sweet potatoes was significantly higher than that of the CK group. The total phenol content of the AA-treated group on days 1 and 5 was significantly higher than that of the CK group (by 41.8% and 11.4%, respectively) (Figure 3A). Over the course of the healing period, the total flavonoid content first increased, then decreased, and then increased. The total flavonoid content in the AA group was extremely significantly higher than that in the CK group (by 24.2%) on day 3 (Figure 3B). Phenols and flavonoids are metabolites of the phenylpropane pathway, and their accumulation is closely related to plant resistance and senescence.

### 3.4. The Contents of Individual Phenolic Acids and Lignin Monomers

Chlorogenic acid, caffeic acid, ferulic acid, sinapic acid, and cinnamic acid are not only the intermediates of the phenylpropane pathway but also the precursors of lignin and SPP synthesis. The chlorogenic acid content in the AA group was extremely significantly higher than that in the CK group after 3 d of healing and significantly higher than that in the CK group on day 7 (Figure 3C). The caffeic acid and cinnamic acid contents of the AA-treated group were extremely significantly higher than the CK group throughout the whole healing period (Figure 3D,G). The ferulic acid content in the AA group was significantly higher than that in the CK group on day 3 (Figure 3E). The sinapic acid content in the AA group was significantly higher than that in the CK group at 1, 5, and 7 d of healing (Figure 3F). *p*-coumaryl alcohol, sinapyl alcohol, and coniferyl alcohol are important monomers that constitute the main components of lignin. The *p*-coumaryl alcohol content of the AA treatment group was extremely significantly higher than the CK group on day 5 (Figure 2D). The sinapyl alcohol content at the wound sites with the AA treatment was extremely significantly higher than that in the CK group after 3 d and 7 d of healing, and significantly higher than control after 5 d of healing (Figure 2E). The coniferyl alcohol content in the AA group was extremely significantly higher than that in the CK group after 1 d of healing and significantly higher than that in the CK group after 3, 5, and 7 d of healing (Figure 2F).

### 3.5. The Contents of MDA and the Permeability of Cell Membranes

Throughout the healing process, the MDA content of the AA-treated and control sweet potatoes showed a trend of first increasing, then decreasing, and then increasing again. However, AA remarkably caused an MDA increase on days 1–7. At 1, 3, 5, and 7 d of healing, the MDA content of the AA-treated sweet potatoes was significantly lower than that of the CK group (by 34.7%, 29.2%, 13.0%, and 29.6%, respectively) (Figure 3H). The cell membrane permeability increased during healing, but the AA treatment delayed the increase compared with the CK group. The cell membrane permeability of the AA-treated sweet potatoes was significantly lower than that of the CK group after 5 and 7 d (Figure 3I). The results showed that AA treatment can effectively reduce the peroxidation degree and protect the structural integrity of the cell membrane at sweet potato wound sites during healing.

### 3.6. The Activities of PAL, C4H, C4L, CAD, and POD

**Figure 4:**
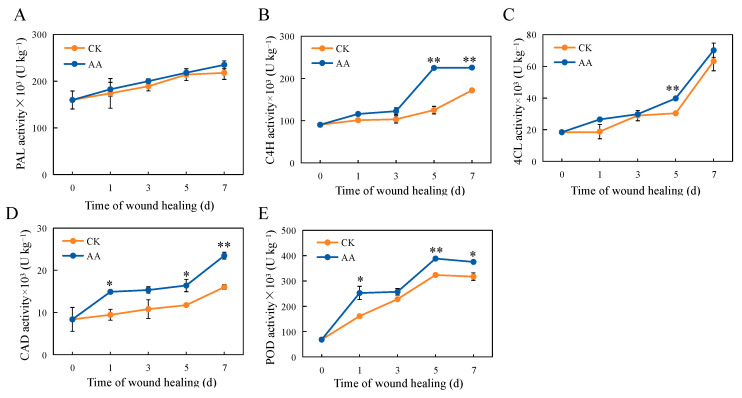
The activity of PAL (**A**), C4H (**B**), 4CL (**C**), CAD (**D**), and POD (**E**) at the sweet potato wound sites during healing. The bars indicate the standard deviation (±SD). The asterisks indicate significant differences between the different treatments at the same time (* *p* < 0.05, ** *p* < 0.01).

PAL, C4H, 4CL, and CAD are key enzymes involved in the phenylpropanoid metabolism pathway, and their activity levels can reflect the strength of phenylpropanoid metabolism. The activity of PAL increased as a result of the AA treatment throughout the whole healing period. After 7 d of healing, the PAL activity of the AA-treated group was 7.3% higher than that of the CK group (Figure 4A). A remarkable increase in C4H activity was observed after the AA treatment, with significant differences of 44.4% and 23.9% on days 5 and 7 of the healing period (Figure 4B). The activity of 4CL increased during the whole healing period, increasing slowly in the early stage of healing and faster in the later stage of healing. The activity of 4CL in the treatment group was stronger than that in the control group. After 5 d of healing, the 4CL activity was increased by about 23.6% when the wounded sweet potatoes were treated with AA (Figure 4C). The activity of the CAD enzyme showed an upward trend throughout the healing period. It could be seen that the CAD activity was significantly enhanced by about 57.7%, 42.1%, 39.5%, and 46.5% at 1, 3, 5, and 7 d of healing (Figure 4D). POD can participate in and regulate the polymerization of lignin in the cell wall by catalyzing the dehydrogenation and polymerization of various lignin monomers, thereby changing the extensibility of the plant cell walls and regulating plant growth [33]. As the healing time was prolonged, the POD activity at the wound sites gradually increased. However, throughout the whole healing period, the POD activity of the AA-treated group was consistently higher than that of the CK group during the same period. It showed a significant increase after 1, 5, and 7 d of healing, which were about 57.0%, 20.1%, and 18.3% higher than the control (Figure 4E).

### 3.7. Analysis of Transcriptomic Data from Sweet Potato Wound Sites

#### 3.7.1. Overview of the RNA Sequencing Data

**Figure 5:**
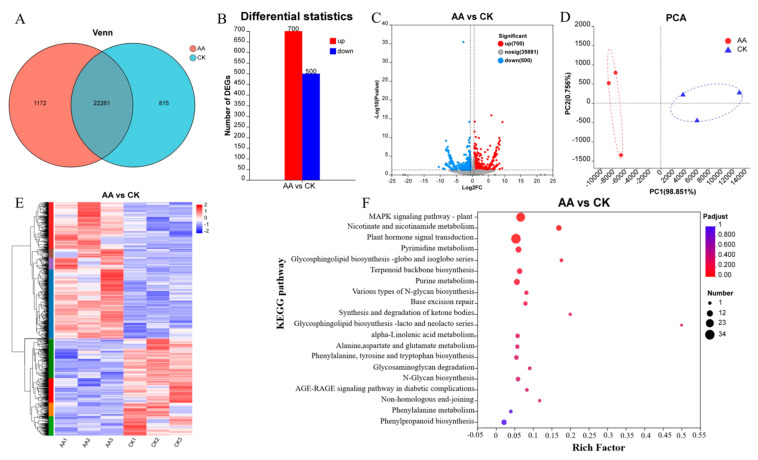
The transcriptomic analysis of sweet potatoes under the different treatments during wound healing: Venn diagram (**A**), quantitative statistics of the differentially expressed genes (**B**), volcano map of the differentially expressed genes (**C**), the principal component analysis (PCA) (**D**), the heatmap cluster analysis of the differentially expressed genes (**E**), and the KEGG enrichment of differentially abundant metabolites (**F**).

To further explain the mechanism of AA promoting sweet potato wound healing, a transcriptional analysis was performed on the AA-treated and CK groups after 3 d of healing. A total of 41.5 Gb of clean data was obtained, with more than 6.13 Gb of clean data for each sample, and the Q30 percentage for the bases was more than 92.7%. A total of 22,261 DEGs were detected between the CK and AA treatments, as shown by the Venn diagram (Figure 5A). A total of 1200 DEGs were detected by applying the screening criteria of a *p* value < 0.05 and an FC ≥ 1.5, including 700 upregulated and 500 downregulated genes (Figure 5B). A volcano plot was generated to visualize the distribution of the DEGs in the different groups (Figure 5C). The FPKMs of all the samples were also analyzed by a principal component analysis (PCA), and the results showed that the AA group and the CK group were clearly separated along the first principal component (PC1), which contributed 98.851% (Figure 5D). The samples from the AA group and the CK group were analyzed by cluster analysis, and the expression of the same gene in different samples was compared (Figure 5E). By a KEGG pathway analysis, the differentially expressed genes were mainly enriched in the MAPK signaling pathway—plant; phytohormone signaling; phenylalanine, tyrosine, and tryptophan biosynthesis; phenylalanine metabolism; and phenylpropane biosynthesis after the AA treatment (Figure 5F).

#### 3.7.2. Related Genes That Play a Role in Healing after AA Treatment

The RNA-seq analysis results showed that two 3-dehydroquinate dehydratase/shikimate dehydrogenase (SKDH) genes and two arogenate/prephenate dehydratase (ADT/PDT) genes were detected. These key genes involved in phenylalanine, tyrosine, and tryptophan biosynthesis are closely related to the synthesis of shikimic acid and phenylalanine. Moreover, one phenolylalanine ammonia lyase, one 4-coumarate-CoA ligase, and three peroxidase genes were also detected. These genes are the key genes in the phenylpropanoid biosynthesis pathway and are closely related to lignin synthesis. The specific levels of up- and downregulation are shown in Table 1.

### 3.8. Analysis by qRT-PCR

**Figure 6:**
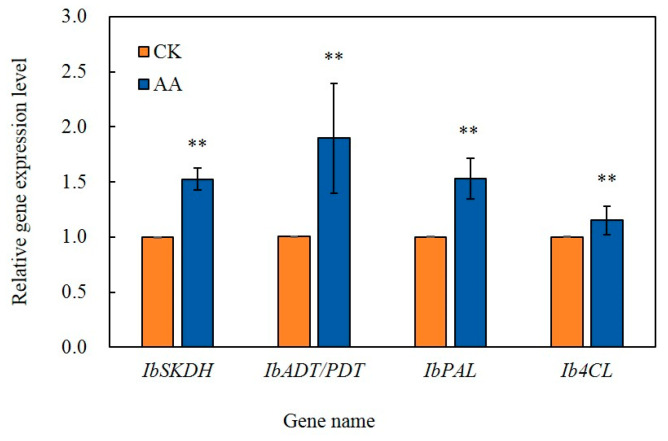
The expression levels of *IbSKDH*, *IbADT/PDT*, *IbPAL*, and *Ib4CL* at the sweet potato wound sites after 3 d of healing. The bars indicate the standard deviation (±SD). The asterisks indicate significant differences between the different treatments (** *p* < 0.01).

*IbSKDH* and *IbADT/PDT* are important genes for the synthesis of shikimic acid and *IbPAL* and *Ib4CL* are key genes in the phenylpropanoid biosynthesis pathway. The qRT-PCR results were consistent with the overall trend in the transcriptome sequencing data, which confirmed that the transcriptome sequencing results were accurate and reliable (Figure 6).

### 3.9. Correlation Analysis

**Figure 7:**
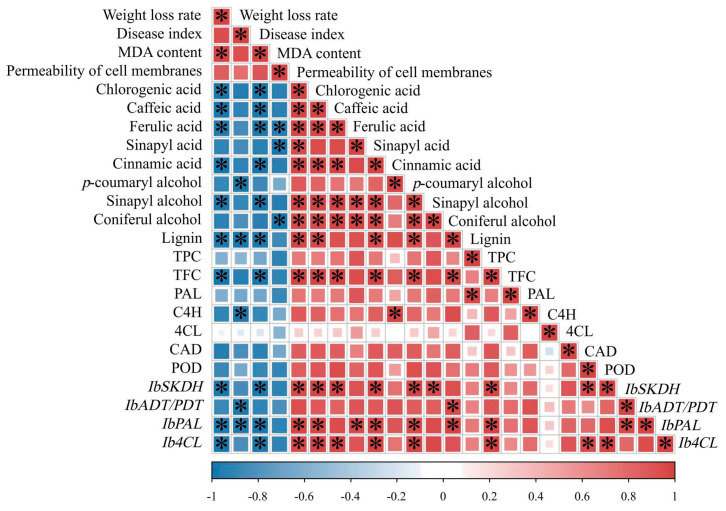
A correlation matrix of the relationships between the phenylpropanoid metabolism pathway and other parameters in sweet potatoes after 20 g L^−1^ of AA treatment during wound healing. Red indicates a strong positive correlation, blue indicates a strong negative correlation, and the asterisks represent significant differences (* *p* < 0.05).

Correlation analyses were carried out for the weight loss rate; disease index; MDA content; permeability of the cell membranes; phenylpropane metabolism-related enzyme levels; lignin, TPC (total phenol content), TFC (total flavonoid content), phenolic acid, and lignin monomer levels; and *IbSKDH*, *IbADT/PDT*, *IbPAL*, and *Ib4CL* expression levels in the healing tissues of sweet potato. The results showed that the lignin content during the healing process was significantly negatively correlated with the weight loss rate, disease index, and MDA content (*p* < 0.05), and the correlation coefficients were −0.97, −0.99, and −0.98, respectively. The lignin content was significantly positively correlated with chlorogenic acid, caffeic acid, cinnamic acid, and sinapyl alcohol contents (*p* < 0.05), and the correlation coefficients were 0.99, 0.96, 0.96, and 0.95, respectively. Moreover, the lignin content was positively correlated with the relative expression of the *IbADT/PDT* and *IbPAL* genes (*p* < 0.05), with correlation coefficients of 0.97 and 0.98. The *IbADT/IbPDT* and *IbPAL* were the key genes in the phenylpropanoid biosynthesis pathway (Figure 7).

## 4. Discussion

This current study found that postharvest AA treatment can effectively maintain the quality of various fruits and vegetables during storage [34,35]. In this study, we found that AA treatment clearly reduced the weight loss rate and disease index of the wounded sweet potatoes, which was similar to the effects of AA in longan [12] and carrot [36]. It may be attributed to the promotion of lignin and SPP formation in the sweet potato healing tissues after the AA treatment, thereby effectively preventing water loss and enhancing resistance to pathogen infections [37]. The healing of sweet potatoes is a process in which the tissue structure is embolized or the cork tissue re-forms the sealing layer and wound periderm after the injury, accompanied by the deposition of SPP and lignin at the wound site to establish a strong barrier to resist any pathogen infection. Similar to the healing processes of potato [38], melon [18], and muskmelon [39], SPP and lignin deposition could be clearly observed at the wound sites.

Phenylpropanoid metabolic is an important secondary metabolic pathway in plants, which can provide the necessary precursors for the formation of lignin and SPP and produce compounds with antifungal and antioxidant activities [40,41]. PAL is the starting and limiting enzyme of the phenylpropane pathway, which can catalyze the production of trans cinnamic acid from L-phenylalanine [42]. The increase in PAL activity can enhance the production of antioxidant defense substances and promote the healing of sweet potato damage [43]. C4H hydroxylates trans cinnamic acid to generate *p*-coumaric acid, which is then converted into caffeic acid, ferulic acid, 5-hydroxyferulic acid, and sinapine through coumarin-3-hydroxylase, caffeic acid-O-methyltransferase, and 5-hydroxyferulacid-O-methyltransferase [42,44]. Then, cinnamic acid, *p*-coumaric acid, caffeic acid, ferulic acid, and sinapic acid are acted upon by 4CL to form cinnamic acid-CoA, *p*-coumaric acid-CoA, caffeic acid-CoA, ferulic acid-CoA, and sinapic acid-CoA, respectively [45]. These phenolic acids-CoA are catalytically converted to *p*-coumarin aldehyde, sinapyl aldehyde, and coniferyl aldehyde under the catalysis of cinnamoyl CoA reductase, and then converted into *p*-coumaryl alcohol, sinapyl alcohol, and coniferyl alcohol through CAD [28,46]. The POD participates in the oxidative polymerization of lignin monomers and the assembly of SPP, playing an important role in the final polymerization of the precursor substances into lignin and SPP [44]. Lignin not only provides structural support for fruit cells but also serves as a physical barrier to defend against pathogen attacks [47,48]. Therefore, based on the results of this study, AA treatment can increase the content of phenolic acids such as chlorogenic acid, caffeic acid, ferulic acid, sinapic acid, and cinnamic acid, as well as the content of sinapyl alcohol, coniferyl alcohol, and *p*-coumaryl alcohol, by activating the key enzymes PAL, C4H, 4CL, and CAD in the phenylpropane pathway at the wound sites of the sweet potato. At the same time, the induction of enhanced POD activity promotes lignin synthesis and SPP assembly, accelerates healing tissue formation, and improves disease resistance.

The RNA-seq analysis revealed that the AA treatment activated phenylalanine tyrosine and tryptophan biosynthesis and the phenylpropane metabolism pathway. The qRT-PCR verification also showed that the AA treatment upregulated the expression of *IbSKDH*, *IbADT/IbPDT*, *IbPAL*, *Ib4CL*, and other genes. *IbSKDH* and *IbADT/IbPDT* are key genes in the phenylalanine, tyrosine, and tryptophan biosynthesis pathway, and *IbSKDH* can catalyze the synthesis of shikimic acid and tryptophan. Shikimic acid is converted to shikimic acid 3-phosphate, branched acid, and prebenzoic acid by shikimate kinase, as well as branched acid synthetase, and further converted to tyrosine and phenylalanine (substrates of phenylpropane metabolism) by the upregulation of *IbTAT* and *IbADT/IbPDT* in this pathway, thus activating the phenylpropane metabolic pathway to synthesize lignin. The PAL, C4H, and 4CL genes are key genes in the lignin biosynthesis pathway. Silencing the PAL gene in tobacco leads to a decrease in the flavonoid and lignin contents and damages plant growth and development [49]. The knockout of the C4H gene in *Arabidopsis thaliana* can inhibit the biosynthesis of flavonoids and lignin [50]. Downregulating the expression of CAD in Arabidopsis thaliana can reduce the content of lignin [51]. The activation of the genes and enzymes involved in lignin synthesis can increase the content of lignin, thus improving plant disease resistance [52,53]. Combined with the effect of the AA treatment on the levels of the key enzymes and products involved in phenylpropane metabolism in sweet potato wound sites, it was further confirmed that this pathway was involved in the healing process. The AA treatment positively regulated the synthesis and accumulation of secondary metabolites related to wound healing by stimulating the transcriptional expression of the phenylpropanoid biosynthesis-related genes. However, the transcriptome results indicated that many metabolic pathways were affected after the AA treatment, and further exploration of which pathway plays a key role should be studied. Previous studies have shown that different sweet potato varieties have varying wound healing efficiencies [54]. Therefore, further verification is needed to determine whether AA treatment can achieve the same effect on sweet potatoes of different varieties and the degrees of damage under the same conditions.

## 5. Conclusions

In this study, it is found that AA treatment can activate phenylpropane metabolism; promote the activities of PAL, C4H, 4CL, CAD, and POD; and increase the content of chlorogenic acid, caffeic acid, ferulic acid, sinapic acid, cinnamic acid, *p*-coumaryl alcohol, sinapyl alcohol, and coniferyl alcohol, as well as that of total phenol, flavonoid, and lignin, at the wound sites of sweet potatoes during healing. With the formation of lignin and SPP, the weight loss rate and disease index were significantly decreased. Moreover, during the healing period, the MDA content was reduced, which could protect the structural integrity of the cell membrane and maintain a better appearance. The results showed that AA played an antioxidant role during the wound healing process. As a safe and low-cost substance, AA is often used in the preservation of fruits and vegetables but is rarely used in healing. This research showed that postharvest AA treatment can accelerate the healing of sweet potatoes.

## Figures and Tables

**Table 1:** Expression levels of DEGs related to wound healing in sweet potato. Asterisks indicate significant differences (* *p* < 0.05, ** *p* < 0.01).

Function	Annotation	Gene ID	Gene Expression (FPKM Value)	
			CK	AA
Phenylalanine, Tyrosine, Tryptophan biosynthesis	3-dehydroquinate dehydratase/shikimate dehydrogenase (SKDH)	itf13g19040.t1	42.7 ± 5.4	75.6 ± 12.3 *
	phosphoribosylanthranilate isomerase (trpF)	itf13g19040.t2	1.2 ± 0.6	2.5 ± 0.3 *
		itf05g14750.t2	1.5 ± 1.7	0.0 ± 0.0
	arogenate/prephenate dehydratase (ADT/PDT)	itf04g31570.t1	71.8 ± 19.3	126.6 ± 11.5 *
	tyrosine aminotransferase (TAT)	itf07g12100.t1	15.8 ± 2.8	25.9 ± 2.4 **
		itf02g12580.t1	0.0 ± 0.0	8.7 ± 5.1 *
Phenylpropanoid Biosynthesis	phenylalanine ammonia-lyase (PAL)	itf06g07070.t1	110.8 ± 30.7	217.9 ± 58.4 *
	feruloyl-CoA 6-hydroxylase (F_6_H)	itf07g02770.t1	14.0 ± 4.6	39.8 ± 35.2
		itf14g07470.t1	1.3 ± 0.8	4.8 ± 3.0
		itf14g07440.t1	1.2 ± 1.0	4.6 ± 2.2
		itf14g07430.t1	1.7 ± 2.0	7.0 ± 1.5 *
	4-coumarate-CoA ligase (4CL)	itf03g10110.t1	15.6 ± 1.9	25.4 ± 3.6 *
	peroxidase (POD)	itf07g05260.t1	0.0 ± 0.0	0.4 ± 0.2 *
		itf14g19200.t1	2.9 ± 0.8	5.7 ± 1.6
		itf09g05830.t1	0.1 ± 0.0	1.1 ± 0.4 **
MAPK signaling pathway—plant	LRR receptor-like serine/threonine-protein kinase FLS2 (FLS2)	itf10g03920.t1	0.6 ± 0.1	2.1 ± 0.9 *
	mitogen-activated protein kinase kinase 3 (MKK3)	itf02g25910.t7	1.1 ± 1.0	0.0 ± 0.0
	transmembrane protein 222 (TMEM222)	itf06g10170.t3	0.0 ± 0.0	0.5 ± 0.4
	ethylene receptor (ETR, ERS)	itf13g20890.t4	16.0 ± 5.2	35.4 ± 7.9 *
		itf04g07670.t1	5.6 ± 2.5	12.4 ± 2.2 *
		itf03g16670.t1	12.3 ± 0.7	19.3 ± 5.0
		itf04g24110.t1	30.9 ± 8.2	59.8 ± 15.9 *
	mitogen-activated protein kinase kinase 9 (MKK9)	itf04g16830.t1	85.6 ± 21.7	139.5 ± 19.3 *
	EIN3-binding F-box protein (EBF1_2)	itf03g20590.t1	26.8 ± 6.7	59.2 ± 6.4 **
		itf03g20570.t1	110.3 ± 21.8	225.3 ± 11.3 **
		itf15g19520.t1	77.9 ± 15.0	132.9 ± 22.7 *
		itf13g01870.t1	33.5 ± 14.5	90.7 ± 24.3 *
	ethylene-responsive transcription factor 1 (ERF1)	itf13g14940.t1	91.1 ± 49.6	184.8 ± 36.1
		itf13g21010.t1	109.6 ± 60.7	249.6 ± 81.4
		itf14g18080.t1	1.3 ± 0.8	4.3 ± 2.4
		itf04g07800.t1	5.5 ± 2.6	13.1 ± 5.2
	5′-3′ exoribonuclease 2 (XRN2, RAT1)	itf02g03750.t3	0.0 ± 0.0	0.4 ± 0.2

## Data Availability

The data presented in this study are available on request from the corresponding author. The data are not publicly available due to privacy restrictions.

## Supplementary Materials

The following supporting information can be downloaded at https://www.mdpi.com/article/10.3390/foods13162569/s1. Figure S1: effects of different AA concentrations on lignin content of sweet potato healing tissues; Figure S2: HPLC chromatogram of the standard substances of chlorogenic acid (1), caffeic acid (2), *p*-coumaryl alcohol (3), coniferyl alcohol (4), sinapyl alcohol (5), *p*-coumaric acid (6), ferulic acid (7), sinapic acid (8), and cinnamic acid (9); Table S1: alignment of the transcriptome sequencing data with the reference genome significance; Table S2: primers for qRT-PCR validation.

## References

[1] Vithu P., Dash S.K., Rayaguru K. (2019). Post-harvest processing and utilization of sweet potato: A review. Food Rev. Int..

[2] Xing K., Li T.J., Liu Y.F., Zhang J., Zhang Y., Shen X.Q., Li X.Y., Miao X.M., Feng Z.Z., Peng X. (2018). Antifungal and eliciting properties of chitosan against *Ceratocystis fimbriata* in sweet potato. Food Chem..

[3] Parmar A., Kirchner S.M., Sturm B., Hensel O. (2017). Pre-harvest curing: Effects on skin adhesion, chemical composition and shelf-life of sweetpotato roots under tropical conditions. East Afr. Agri. For. J..

[4] Huang C.L., Liao W.C., Chan C.F., Lai Y.C. (2014). Storage performance of Taiwanese sweet potato cultivars. J. Food Sci. Technol..

[5] Wang C.X., Chen L., Peng C.L., Shang X.Q., Lv X.L., Sun J., Li C., Wei L., Liu X.L. (2020). Postharvest benzothiazole treatment enhances healing in mechanically damaged sweet potato by activating the phenylpropanoid metabolism. J. Sci. Food Agr..

[6] Yang R., Han Y., Han Z., Ackah S., Li Z., Bi Y., Yang Q., Prusky D. (2020). Hot water dipping stimulated wound healing of potato tubers. Postharvest Biol. Technol..

[7] Xin Q., Liu B., Sun J., Fan X., Li X., Jiang L., Hao G., Pei H., Zhou X. (2022). Heat shock treatment promoted callus formation on postharvest sweet potato by adjusting active oxygen and phenylpropanoid metabolism. Agriculture.

[8] Ji C.Y., Kim Y.H., Lee C.J., Su U.P., Lee H.U., Kwak S.S., Kim H.S. (2022). Comparative transcriptome profiling of sweetpotato storage roots during curing-mediated wound healing. Gene.

[9] Tu Y.J., Njus D., Schlegel H.B. (2017). A theoretical study of ascorbic acid oxidation and HOO^·^/O_2_^−·^ radical scavenging. Org. Biomol. Chem..

[10] Sakimin S.Z., Patrie S.S., Juraimi A.S., Alam M.A., Aslani F. (2017). Application of ascorbic acid in maintenance of minimally processed product quality of jackfruit (*Artocarpus heterophyllus* Lam). Bangl. J. Bot..

[11] Zhao H., Nie K., Zhou H., Yan X., Zhan Q., Zheng Y., Song C.P. (2020). ABI5 modulates seed germination via feedback regulation of the expression of the *PYR/PYL/RCAR* ABA receptor genes. New Phytol..

[12] Liu J., Lin Y., Lin H., Lin M., Fan Z. (2021). Impacts of exogenous ROS scavenger ascorbic acid on the storability and quality attributes of fresh longan fruit. Food Chem. X.

[13] Xu D., Chen C., Zhou F., Liu C., Tian M., Zeng X., Jiang A. (2022). Vacuum packaging and ascorbic acid synergistically maintain the quality and flavor of fresh-cut potatoes. LWT Food. Sci. Technol..

[14] Antonova G.F., Chaplygina I.A., Varaksina T.N., Stasova V.V. (2005). Ascorbic acid and xylem development in trunks of the Siberian larch trees. Russ. J. Plant Physiol..

[15] Kärkönen A., Fry S.C. (2006). Effect of ascorbate and its oxidation products on H_2_O_2_ production in cell-suspension cultures of *Picea abies* and in the absence of cells. J. Exp. Bot..

[16] Smirnoff N. (2000). Ascorbic acid: Metabolism and functions of a multi-facetted molecule. Curr. Opin. Plant Biol..

[17] Çoban G.A., Aras S. (2023). Effects of ascorbic and oxalic acids on cucumber seedling growth and quality under mildly limey soil conditions. Gesunde Pflanz..

[18] Xue S., Bi Y., Ackah S., Li Z., Li B., Wang B., Wang Y., Li Y., Prusky D. (2023). Sodium silicate treatment accelerates biosynthesis and polymerization of suberin polyaliphatics monomers at wounds of muskmelon. Food Chem..

[19] Zhu Y., Zong Y., Liang W., Kong R., Gong D., Han Y., Li Y., Bi Y., Prusky D. (2022). Sorbitol immersion accelerates the deposition of suberin polyphenolic and lignin at wounds of potato tubers by activating phenylpropanoid metabolism. Sci. Hortic..

[20] Wu J., Pang L., Zhang X., Lu X., Yin L., Lu G., Cheng J. (2022). Early discrimination and prediction of *C. fimbriata*-infected sweetpotatoes during the asymptomatic period using electronic nose. Foods.

[21] Soteriou G.A., Kyriacou M.C., Siomos A.S., Gerasopoulos D. (2014). Evolution of watermelon fruit physicochemical and phytochemical composition during ripening as affected by grafting. Food Chem..

[22] He X.X., Zhang T.T., Wang F.L., Guan W.Q., Lin Q., Sun X.L. (2024). The combination treatment of low voltage electrostatic field and Ultraviolet-C could accelerate the process of wound healing of potato tubers. LWT Food. Sci. Technol..

[23] Fugate K.K., Ribeiro W.S., Lulai E.C., Deckard E.L., Finger F.L. (2016). Cold temperature delays wound healing in postharvest sugarbeet roots. Front. Plant Sci..

[24] Torres A., Basurto F., Navarro-Ocana A. (2019). Quantitative analysis of the biologically active compounds present in leaves of mexican sweet potato accessions: Phenols, flavonoids, anthocyanins, 3,4,5-tri-caffeoylquinic acid and 4-feruloyl-5-caffeoylquinic acid. Plant Food Hum. Nutr..

[25] Xie Y., Zheng Y., Dai X., Wang Q., Cao J., Xiao J. (2015). Seasonal dynamics of total flavonoid contents and antioxidant activity of Dryopteris erythrosora. Food Chem..

[26] Ayaz F.A., Hayirlioglu-Ayaz S., Gruz J., Novak O., Strnad M. (2005). Separation, characterization, and quantitation of phenolic acids in a little-known blueberry (*Vaccinium arctostaphylos* L.) fruit by HPLC-MS. J. Agric. Food Chem..

[27] Jiang H., Wang Y., Li C., Wang B., Ma L., Ren Y., Bi Y., Li Y., Xue H., Prusky D. (2020). The effect of benzo-(1,2,3)-thiadiazole-7-carbothioic acid S-methyl ester (BTH) treatment on regulation of reactive oxygen species metabolism involved in wound healing of potato tubers during postharvest. Food Chem..

[28] Qu G.F., Wu W.N., Ba L.J., Ma C., Ji N., Cao S. (2022). Melatonin enhances the postharvest disease resistance of blueberries fruit by modulating the jasmonic acid signaling pathway and phenylpropanoid metabolites. Front. Chem..

[29] Wang H., Kou X., Wu C., Fan G., Li T. (2020). Methyl jasmonate induces the resistance of postharvest blueberry to gray mold caused by *Botrytis cinerea*. J. Sci. Food Agric..

[30] Li H., Suo J.T., Han Y., Liang C.Q., Jin M.J., Zhang Z.K., Rao J.P. (2017). The effect of 1-methylcyclopropene, methyl jasmonate and methyl salicylate on lignin accumulation and gene expression in postharvest ‘Xuxiang’ kiwifruit during cold storage. Postharvest Biol. Technol..

[31] Li S.E., Xu Y.H., Bi Y., Zhang B., Shen S.L., Jiang T.J., Zheng X.L. (2019). Melatonin treatment inhibits gray mold and induces disease resistance in cherry tomato fruit during postharvest. Postharvest Biol. Technol..

[32] Li C., Zhang C., Liu J., Qu L., Ge Y. (2023). L-glutamate maintains the quality of apple fruit by mediating carotenoid, sorbitol and sucrose metabolisms. J. Sci. Food Agric..

[33] Luo Y., Zeng K., Ming J. (2012). Control of blue and green mold decay of citrus fruit by *Pichia membranefaciens* and induction of defense responses. Sci. Hortic..

[34] Yan S., Luo Y., Zhou B., Ingram D.T. (2017). Dual effectiveness of ascorbic acid and ethanol combined treatment to inhibit browning and inactivate pathogens on fresh-cut apples. LWT Food Sci. Technol..

[35] Zhao X., Guo S., Ma Y., Zhao W., Wang P., Zhao S., Wang D. (2022). Ascorbic acid prevents yellowing of fresh-cut yam by regulating pigment biosynthesis and energy metabolism. Food Res. Int..

[36] Xylia P., Clark A., Chrysargyris A., Romanazzi G., Tzortzakis N. (2019). Quality and safety attributes on shredded carrots by using Origanum majorana and ascorbic acid. Postharvest Biol. Technol..

[37] Machado A., Pereira H., Teixeira R.T. (2013). Anatomy and development of the endodermis and phellem of *Quercus suber* L. roots. Microsc. Microanal..

[38] Yang R., Han Y., Zhang X., Wang Q., Zheng X., Wang Y., Li Y., Prusky D., Bi Y. (2023). Ferulic acid treatment enhances the synthesis, transport and deposition of suberin polyaliphatic monomers on potato tuber wounds. Postharvest Biol. Technol..

[39] Wang B., Jiang H., Bi Y., He X., Wang Y., Li Y., Zheng X., Prusky D. (2019). Preharvest multiple sprays with sodium nitroprusside promote wound healing of harvested muskmelons by activation of phenylpropanoid metabolism. Postharvest Bio. Technol..

[40] Dong N.Q., Lin H.X. (2021). Contribution of phenylpropanoid metabolism to plant development and plant-environment interactions. J. Integr. Plant Biol..

[41] Cui P., Li Y.X., Cui C.K., Huo Y.R., Lu G.Q., Yang H.Q. (2020). Proteomic and metabolic profile analysis of low-temperature storage responses in *Ipomoea batata* Lam. tuberous roots. BMC Plant Biol..

[42] Vogt T. (2010). Phenylpropanoid biosynthesis. Mol. Plant.

[43] Yin J., Bai S., Wu F., Lu G., Yang H. (2012). Effect of nitric oxide on the activity of phenylalanine ammonia-lyase and antioxidative response in sweetpotato root in relation to wound-healing. Postharvest Bio. Technol..

[44] Arrieta-Baez D., Stark R.E. (2006). Modeling suberization with peroxidase-catalyzed polymerization of hydroxycinnamic acids: Cross-coupling and dimerization reactions. Phytochemistry.

[45] Sutela S., Hahl T., Tiimonen H., Aronen T., Ylioja T., Laakso T., Saranpää P., Chiang V., Julkunen-Tiitto R., Häggman H. (2014). Phenolic compounds and expression of 4CL genes in silver birch clones and Pt4CL1a lines. PLoS ONE.

[46] Barros J., Serk H., Granlund I., Pesquet E. (2015). The cell biology of lignification in higher plants. Ann. Bot..

[47] Khan M.K.U., Zhang X., Ma Z., Huang M., Yang C., Wang X., Peng J. (2023). Contribution of the LAC genes in fruit quality attributes of the fruit-bearing plants: A comprehensive review. Int. J. Mol. Sci..

[48] Mahto R., Das M. (2014). Effect of gamma irradiation on the physico-mechanical and chemical properties of potato *(Solanum tuberosum* L.), cv. ‘Kufri Sindhuri’, in non-refrigerated storage conditions. Postharvest Biol. Technol..

[49] Korth K.L., Blount J.W., Chen F., Rasmussen S., Lamb C., Dixon R.A. (2001). Changes in phenylpropanoid metabolites associated with homology-dependent silencing of phenylalanine ammonia-lyase and its somatic reversion in tobacco. Physiol. Plant..

[50] Schilmiller A.L., Stout J., Weng J.K., Humphreys J., Ruegger M.O., Chapple C. (2009). Mutations in the *cinnamate 4-hydroxylase* gene impact metabolism, growth and development in *Arabidopsis*. Plant J..

[51] Thévenin J., Pollet B., Letarnec B., Saulnier L., Gissot L., Maia-Grondard A., Lapierre C., Jouanin L. (2011). The simultaneous repression of CCR and CAD, two enzymes of the lignin biosynthetic pathway, results in sterility and dwarfism in *Arabidopsis thaliana*. Mol. Plant.

[52] Veronico P., Paciolla C., Pomar F., De Leonardis S., García-Ulloa A., Melillo M.T. (2018). Changes in lignin biosynthesis and monomer composition in response to benzothiadiazole and root-knot nematode *Meloidogyne incognita* infection in tomato. J. Plant Physiol..

[53] Li S.S., Chang Y., Li B., Shao S.L., Zhang Z.Z. (2020). Functional analysis of 4-coumarate: CoA ligase from *Dryopteris fragrans* in transgenic tobacco enhances lignin and flavonoids. Genet. Mol. Biol..

[54] van Oirschot Q.E.A., Rees D., Aked J., Kihurani A. (2006). Sweetpotato cultivars differ in efficiency of wound healing. Postharvest Biol. Technol..

