# Supplementary material for: Physiological–Biochemical Characteristics and a Transcriptomic Profiling Analysis Reveal the Postharvest Wound Healing Mechanisms of Sweet Potatoes under Ascorbic Acid Treatment
Source: Foods. 2024 Aug 17;13(16):2569. doi: 10.3390/foods13162569 (PMC13159385; doi:10.3390/foods13162569)
Supplement: Supplementary file 1 [file foods-13-02569-s001.zip › foods-3128684 Supplementary materials.pdf]

Supplementary materials

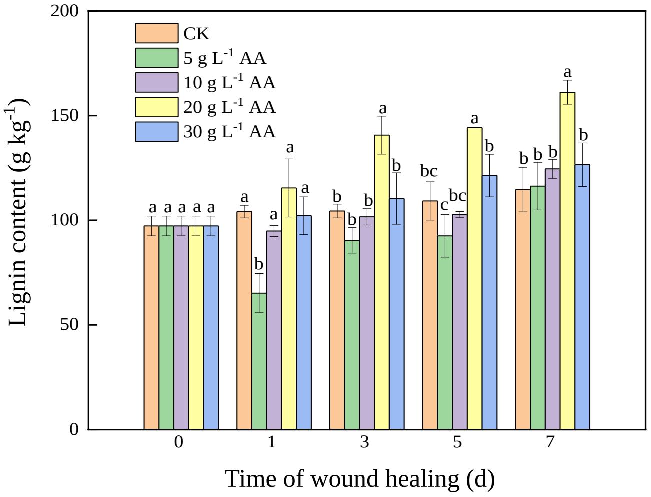

Figure S1. Effects of different AA concentrations of on lignin content at sweet potato healing tissues. Notes: Vertical bars indicate standard deviation (±SD). Different letters indicate significant differences among different treatments at the same time each day (*P*<0.05).

Results: Taking the lignin content as an index, the artificial injured sweet potato was treated with AA of 0, 5, 10, 20, 30 g L^{-1} respectively, and the effect of AA treatment on the healing ability of sweet potato was evaluated. The experimental results are as shown by Figure S1. With the increase of time, the increased amount of lignin in the wound site of sweet potato in 20 g L^{-1} AA treatment group was the most obvious. On the 3, 5 and 7 day, the lignin content of 20 g L^{-1} AA-treated group was significantly higher than other groups ($P < 0.05$). Therefore, in the follow-up experiment, 20 g L^{-1} AA was used to treat the healing of postharvest sweet potatoes to further explore the regulation mechanism of AA treatment on the healing ability.

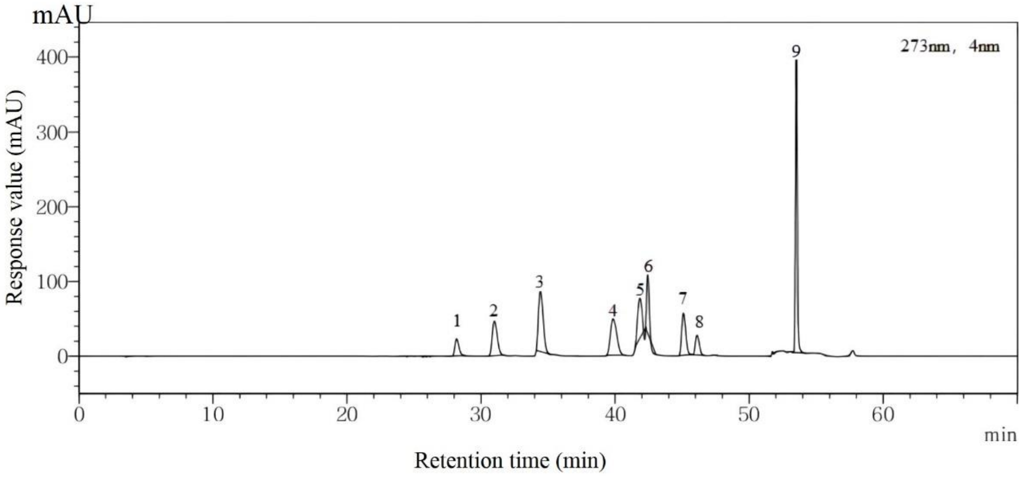

Figure S2. HPLC chromatogram of the standard substance of chlorogenic acid (1), caffeic acid (2), *p*-coumaryl alcohol (3), coniferyl alcohol (4), sinapyl alcohol (5), *p*-coumaric acid (6), ferulic acid (7), sinapic acid (8), cinnamic acid (9).

Table S1. Alignment of the transcriptome sequencing data with the reference genome significant

sample	repeat	Total reads	Total mapped	Multiple mapped
CK	1	47510016	36892417(77.65%)	1602991(3.37%)
	2	48010672	36448272(75.92%)	1617745(3.37%)
	3	44005452	33940715(77.13%)	1484696(3.37%)
AA	1	41750664	32804990(78.57%)	1297931(3.11%)
	2	55680040	43668028(78.43%)	1704529(3.06%)

3	45745212	35617991(77.86%)	1447159(3.16%)

Table S2. Primers for qRT-PCR validation

Gene name	Gene ID	Forward primer sequence	Reverse primer sequence
tubulin	/	CAACTACCAGCCACCAACTGT	CAAGATCCTCACGAGCTTCAC
IbSKDH	itf13g19040.t1	CGAGGAAGAACCCGACTC	CCCAGCTCCATTGCTAAA
IbADT/PDT	itf04g31570.t1	GTCGGTTCCAATCGTCTCG	GCCGCTCTTCTGAACATCC
IbPAL	itf06g07070.t1	TCCTTAGGGCTGATCTCGG	CCACTTGGCTCACGCTGT
Ib4CL	itf03g10110.t1	GAGGATTTCTCGGTGGTC	TTAGGTATAGGTTCGGGTTCT
